# Intraoperative Monitoring of Neuromuscular Blockade

**DOI:** 10.3390/life13051184

**Published:** 2023-05-15

**Authors:** Cyrus Motamed

**Affiliations:** Institut de Cancérologie Gustave Roussy, 94080 Villejuif, France; cyrus.motamed@gustaveroussy.fr

**Keywords:** neuromuscular monitoring, neuromuscular blockade, residual neuromuscular blockade, peripheral nerve stimulator, quantitative monitoring, sugammadex, calibration

## Abstract

There is a global trend of new guidelines highly recommending quantitative neuromuscular monitoring in the operating room. In fact, it is almost certain that quantitatively monitoring the depth of intraoperative muscle paralysis may permit the rational use of muscle relaxants and avoid some of the major related complications, namely postoperative pulmonary complications. A specific culture related to this issue is necessary to integrate quantitative monitoring of muscle relaxants as part of a major monitoring entity in anesthetized patients. For this purpose, it is necessary to fully understand the physiology, pharmacology and concept of monitoring as well as the choice of pharmacological reversal, including the introduction of sugammadex a decade ago.

## 1. Introduction

Neuromuscular blocking agents (NMBAs) are administered during anesthesia or in intensive care to facilitate endotracheal intubation and/or to improve surgical conditions or ventilation. Neuromuscular blockade or paralysis should be monitored for all patients who receive NMBAs during anesthesia, in order to optimize the dosing of NMBAs and the time of administration of reversal agents if necessary and to assess the time pattern of recovery [1]. Quantitative objective neuromuscular monitoring is now recommended in many anesthetic societies [2,3] since clinical assessment or simple nerve stimulators do not accurately reflect the degree of neuromuscular paralysis and this discrepancy might yield residual neuromuscular blockade before awakening the patient. Indeed, in the last analysis of the European Society of Anaesthesiology and Intensive Care (ESAIC), the incidence of residual paralysis when neuromuscular monitoring was performed clinically was 0.3%, while this incidence was significantly lower during quantitative objective monitoring 0.1%. The most important time for monitoring neuromuscular blockade is during the time of recovery; however, monitoring is useful in all sequences of anesthesia starting from induction, continuing during maintenance and terminating at the time of awakening. This narrative review will comment on recent literature and guidelines on this issue.

## 2. Mechanism of Neuromuscular Transmission at the Neuromuscular Junction

**Figure 1:**
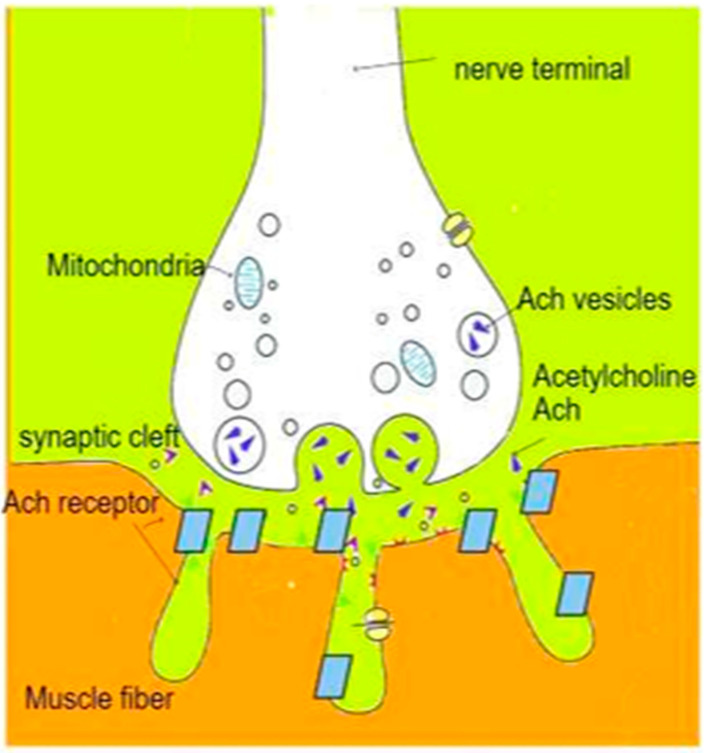
Simplified physiology of neuromuscular junction.

**Figure 2:**
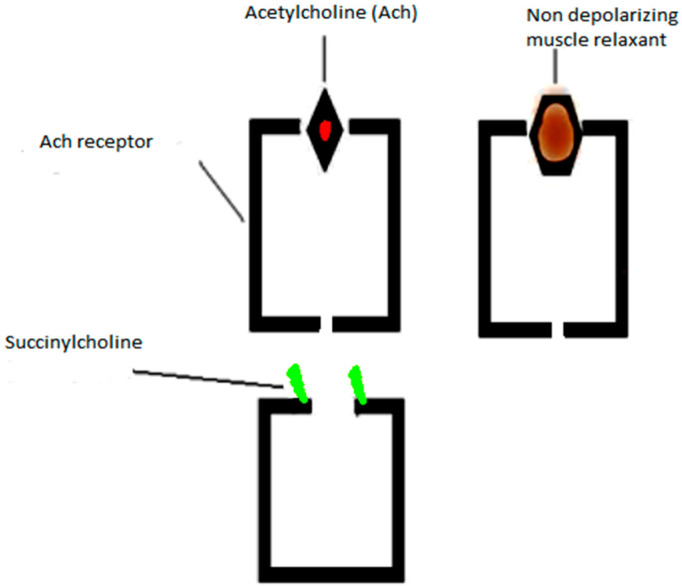
Acetylcholine receptor and the site of action of non-depolarizing and depolarizing muscle relaxants (NMDA). NMDA counteracts the action of Ach on the postsynaptic membranes, thus preventing its depolarizing action and excluding the possibility of exciting muscle fibers, while depolarizing relaxants (succinylcholine) cause initial activation (depolarization) of the receptor and subsequent prolonged, stable blockade.

The motor unit is a motor neuron connected to a muscle via the neuromuscular junction. One nerve fiber is connected usually to a maximum of 2000 muscle fibers [4]. The neuromuscular junction is the point of communication between a motor neuron and a muscle fiber [5]. The action potential reaches the motor nerve terminal and yields depolarization. This depolarization induces a stream of calcium ions into the motor nerve axon, permitting the adhesion of acetylcholine (Ach) vesicles to the presynaptic membrane [6,7]. The Ach molecules which are released in the postsynaptic cleft bind to the postsynaptic receptors and generate an end plate potential (EPP). When the EPP reaches a threshold, the sodium conductance of the muscle membrane increases, which triggers muscle action potential; this transmission is ended by the mobilization of acetylcholine at the synapse and rapid dissociation by acetylcholinesterase (ACHE) (Figure 1). The depolarizing and non-depolarizing muscle relaxant sites of action are displayed in Figure 2.

## 3. Rationale for Monitoring Neuromuscular Blockade

The main indication of neuromuscular blockade monitoring remains the detection of residual neuromuscular blockade. Residual neuromuscular blockade is the remaining weakness of muscles after the administration of neuromuscular blockade and is reported to be related to postoperative pulmonary complications [8]. The definition of residual paralysis is now quantitative and is defined by a ratio of train of four (TOFR) stimulation below 0.9. Clinical signs of residual neuromuscular blockade include: muscle weakness, swallowing difficulty, pneumonia and aspiration, respiratory distress including desaturation, and airway obstruction. The projection of muscle paralysis or muscle weakness in the context of neuromuscular monitoring is the presence of fatigue or fade. It is a decreased contraction force after several consecutive contractions. This represents the blockade mediated by the nicotinic presynaptic receptor [9]. Neuromuscular monitoring of anesthetized patients should start at the induction of anesthesia to facilitate tracheal intubation until the end of recovery and extubation as there is an intense variability in the pharmacodynamics of muscle relaxants including interaction with other anesthetic agents making clinical course of the onset and offset of their effect almost totally unpredictable. While monitoring the recovery of muscle relaxation is extensively recommended and reported, full-time monitoring including induction, maintenance and recovery is not as popular, and sparse data exist. The latest French guidelines on muscle relaxants in 2020 [1] recommended intraoperative monitoring of neuromuscular paralysis until the end of recovery. More recently, the ESAIC and American Society of Anesthesiology (ASA) published new guidelines for the intraoperative handling of neuromuscular blockade [3]. The guidelines from these two major anesthesia societies strongly recommend the routine use of quantitative neuromuscular monitoring, which is a significant boost for this issue. Indeed, objective monitoring of neuromuscular blockade at the induction of anesthesia (onset of blockade) permits the detection of the appropriate time of intubation if muscle relaxants are used, while monitoring the depth of neuromuscular block during maintenance of anesthesia permits administration or reinjection of an adequate dose, avoiding over- or underdosage of the relaxants. 

## 4. Different Types of Neuromuscular Stimulation

### 4.1. Single Twitch or Single Stimuli

This mode of stimulation consists of repetitive supramaximal stimuli for a period of 0.2 ms at regular intervals [10]. When 75% of postsynaptic nicotinic receptors, are occupied the twitch response will be depressed. The most used time interval between each stimulation is 1 s. This stimulation is mostly useful during the onset of blockade, but it is a poor indicator of profound paralysis and could reflect normal contraction while an important weakness is present. It also has a major limitation, which is a need for measurement of controlled twitch; however, the current trend is now focusing on train of four stimulation, and this mode of stimulation will probably be progressively abandoned. 

### 4.2. Train of Four (TOF) Stimulation

This is the most commonly used pattern of stimulation. It was designed by Ali et al. in 1970 [11] in order to develop a clinical tool to evaluate neuromuscular blockade in patients under anesthesia which did not require a control response before administration of muscle relaxants. This pattern is composed of four stimuli at 2 Hz separated by 0.5 ms, and the minimal pattern of repetition is 10 s; however, during maintenance of paralysis, this interval can be widened to long as several minutes. The comparison of the fourth response (T4) to the first response (T1) is the basis of the TOFR, as fade appears first on the fourth twitch as soon as muscle relaxants are acting at the presynaptic level. Progressively, if a full dose is given, T4 disappears, followed by T3 to T1; during recovery, T1 reappears first and T4 reappears last. It should be noticed that the fade pattern does not appear in the presence of succinylcholine, which is a depolarizing muscle relaxant; however, in the case of cholinesterase deficiency, a deep block can appear and recovery could emulate a non-depolarizing picture.

### 4.3. Double Burst Stimulation (DBS)

This method was initially designed by Engbaek et al. [12] to detect better small residual neuromuscular paralysis once the non-quantitative TOFR could not detect it; indeed, the fade detected by this method is more perceptible to the naked eye or tactilely in comparison to TOF. The method consists of two bursts separated by 750 milliseconds (ms), with three impulses in each burst separated by 20 ms. The absence of visual or tactile fade during this mode of stimulation does not exclude residual paralysis. The DBS fade can also be quantified and displayed with modern monitors.

### 4.4. Tetanus

In this mode, 50 to 200 Hz supramaximal stimulation is used for 5 s, while the contraction in healthy skeletal muscle is maintained, as in pure depolarizing block. Fade appears after administration of muscle relaxants or in a phase 2 depolarizing block while using 50 Hz frequency which is very sensitive [10,13]. If a higher frequency is used, fatigue may appear in normal muscle without a neuromuscular blocking agent. This mode of stimulation is seldom indicated and should be used in anesthetized patients only as it is extremely painful.

### 4.5. Post-Tetanic Count (PTC)

This method was designed to refine the magnitude of deep neuromuscular blockade. The PTC can be used only if the TOF count is null. In this setting, a 5 s 50 Hz tetanus is delivered and followed 3 s later by 10 to 20 single twitch stimuli. In the very early stages of recovery, a number of responses to the single twitch may be observed. The number of responses is inversely proportional to the first response of TOF and is a valuable “add-in” to predict approximate recovery; this phenomenon is the post-tetanic facilitation, as after the 5 s tetanus, a number of acetylcholine receptors yield an increased response to the TOF stimulation. However, the repetition of this mode of stimulation every 3 min does not affect the final 90% TOF recovery [14]. The PTC is mostly effective in surgery necessitating deep paralysis. This mode of stimulation is also a better indicator of early diaphragmatic recovery in comparison to corrugator supercilia [15]. PTC is also useful to define the exact moment of sugammadex administration [16].

A summary of different types of stimulation is displayed in Table 1.

## 5. Different Sites of Neuromuscular Monitoring 

Different muscles act differently in response to the administration of muscle relaxants. The onset of neuromuscular blockade in central muscles such as the diaphragm is faster than that in peripheral muscles such as the adductor pollicis. Neuromuscular block has shorter duration and faster recovery in laryngeal muscles in comparison to the adductor pollicis [17,18]. 

Monitoring neuromuscular blockade can be performed in multiple sites by stimulating different nerves and measuring the response of the muscles innervated by the relevant nerve. This site should have easy access for the anesthesia providers during the surgery or procedures. The evoked response of the ulnar nerve at the adductor pollicis is the gold standard in anesthesia. This site cannot be used if it is inaccessible in certain types of surgery or procedures or if it has previous nerve or muscle damage that could alter the evoked response to stimulation, such as muscle paralysis which may yield elevated TOFR as a consequence of upregulation of Ach receptors. 

The second site is the facial nerve group orbicularis oculi and corrugator supercilii; indeed, despite their proximity, these two muscles do not have the same pharmacodynamics profile. The orbicularis oculi is very similar to the adductor pollicis for recovery, while the profile of the corrugator supercilii is mostly the same as that of laryngeal muscles for predicting the optimal time for intubation [19]. Not all muscles have the same sensitivity to the effects of non-depolarizing muscle relaxants. More than 95% of published studies use ulnar nerve stimulation with evaluation of the adductor pollicis contraction given the ease of access but also the possibility of quantification of the muscle response of this muscle. The reference intraoperative stimulation remains the evaluation of the response of the adductor pollicis after stimulation by train of four. Presently, there is no quantitative monitor specially marketed for muscles around the eyes; subsequently, it is recommended to switch as soon as possible to objective monitoring of the adductor pollicis when surgical conditions allow it, in particular for recovery [20]. In the recent guidelines from ESAIC and ASA, monitoring eye muscles is no longer recommended, and the only recommended site for monitoring intraoperative paralysis is the adductor pollicis. 

Monitoring sites in animals may also differ. In pigs, the acceleration transducer can be placed between the third and fourth digits, the cathode placed 10–12 cm from the elbow joint and the anode 4–6 cm proxomedially [21]. In ponies, the peroneus superficialis is reported in association with the ventral buccal branch of the facial nerve, and also the radial nerve was identified on the non-dependent thoracic limb by palpation of the groove between the lateral extensor digitorum and the lateral ulnaris muscles 4 cm distal to the lateral tuberosity of the radius [22]. For cats, the monitoring site reported in a recent study is the peroneal nerve with electrodes placed 1 cm apart in the lateral tibial area [23]. 

## 6. Methods of Neuromuscular Monitoring

### 6.1. Clinical Evaluation

Introduced decades ago upon the use of muscle relaxants in clinical practice, the clinical evaluation included respiratory parameters and muscle strength measures such as a head lift test for 5 s. All of these evaluations are unreliable, especially when the level of paralysis is in the course of recovery or the patient is still recovering from other agents of anesthesia which might increase the level of sedation and contribute to a lack of cooperation [24]. 

### 6.2. Qualitative Evaluation

This evaluation is performed by peripheral nerve stimulators by means of visual and tactical assessments of the evoked response. Currently, all simple nerve stimulators can provide all types of stimulation. Detection of fade is the basic principle in this type of assessment. TOF stimulation, double burst stimulation and tetanus 50 Hz are adequate tests to detect residual paralysis but do not permit anesthesia providers to assess qualitatively up to 0.6–0.9 TOF ratio. Monitoring of neuromuscular blockade using a simple nerve stimulator is more precise than the simple clinical assessment [25] which uses pharmacological data; nevertheless, simple monitoring does not allow an estimation of the exact state of neuromuscular paralysis, especially when it is located in intermediate values; visual and tactile estimation are not as accurate, and evaluation remains usually in a lower range compared to quantified instrumental monitoring. Blum et al. [26] recently compared qualitative monitoring (tactile) versus quantitative monitoring (accelerography) in abdominal and gynecological surgery and found quantitative monitoring permitted the use of a lower quantity of muscle relaxants which putatively might decrease the incidence of residual neuromuscular blockade. It should be remembered that simple qualitative monitoring using a nerve stimulator does not yet prevent adverse respiratory effects [27] or decrease the dose of relaxant given [28] but remains preferable compared to the total absence of monitoring [29]. In addition, the monitoring of eye muscle groups by visual estimation is easier with a simple monitor because the other quantitative monitors currently on the market are not designed for this site. Whenever possible, objective instrumental monitoring should be used during intraoperative muscle relaxation, which is suggested by a high level of evidence [2,3]. Calibration before administrating muscle relaxants usually guarantees better accuracy of the results, particularly in moderate neuromuscular blockade [30]. If, however, the calibration is not possible, it is necessary to increase vigilance, taking into account all the clinical parameters in the interpretation of the results. If available, the measurements can be double checked with a portable device that does not need calibration such as the TOFscan (Dräger, Lübeck, Germany) accelerometer [31]. There are also other monitoring sites available such as flexor hallucis at the toe and first dorsal interosseous and the abductor digiti minimi on the hand, but some inconsistencies exist in their reliability in routine practice. 

## 7. Techniques and Technologies Commercially Available for Quantitative Monitoring of Neuromuscular Blockade 

### 7.1. Acceleromyography

Acceleromyography is based on second Newton’s law of motion, force = mass × acceleration. When mass is constant, acceleration is directly proportional to force. As the force acting upon an object is increased, the acceleration of the object is increased. As the mass of an object is increased, the acceleration of the object is decreased. In this setup, an acceleration transducer, consisting of a piezoelectric sensor, reflects the force of contraction. Whenever the piezoelectric sensor is displaced, a current voltage is generated; therefore, the evoked response to stimulation generates an electric signal. The signal is analyzed, transformed and recorded in a monitoring display. Many commercial models are available, and this is probably the most popular technique for quantitative neuromuscular monitoring. One difficulty with this mode of monitoring is that in many cases the thumb does not return to its initial position after the first stimulation, and therefore the initial measurement often displays a TOFR greater than 1. In order to correct this, the application of a minimal preload, such as the TOFwatch (Organon Ireland) hand adapter, is reported to increase the accuracy of the measurements. If the minimal preload or a hand adapter is not used, normalization of the measurements is necessary; therefore, a correction should be performed by comparing the measurements to the initial value in order to eliminate residual neuromuscular blockade. This technique largely contributed to much of the knowledge in the recent research on residual neuromuscular blockade and monitoring [19,32,33,34,35]. The calibration issue is also debated with this technique, and indeed, an initial calibration permits accuracy and precision in the clinical decision at the end of recovery. The absence of calibration might be corrected by double checking eventually by another device not needing calibration such as a TOFScan accelerograph [31] or additional clinical vigilance in the interpretation of data [36].

### 7.2. Electromyography (EMG)

This technique measures the electrical response of the evoked response after nerve stimulation; here again, the electrical response of the muscle is proportional to the force of contraction. The main advantage of this technique is that the immobilization of the arm is not mandatory, and therefore this technique can suit all types of surgery as free movement and muscle immobility are not required. Some interferences with other electrical devices in the operating room might alter the true response; nevertheless, EMG remains a reliable technique for monitoring. The recently commercially available TetraGraph (Senzime, Sweden) is reported to be interchangeable with an acceleromyographic monitor such as TOFSscan or TOF watch SX [37,38].

### 7.3. Kinemyography (KMG)

KMG has been available for at least 25 years, mainly integrated into the Datex anesthesia station (GE Datex Ohmeda, Madison, WI, USA). The principle is based on measuring an electric signal yielded by the movement of the evoked response muscle which distorts a plastic sensor (piezoelectric effect) that is applied into the groove of the thumb and forefinger and can be secured with tape. In addition, accuracy and precision may increase when it is attached to an arm board. We found that its agreement with MMG for scientific purposes might be limited with unacceptably wide limits of agreement [39] in clinical circumstances. However, it can be used to detect the onset time of muscle relaxation and recovery of NMB in acceptable agreement in comparison to a force transducer. One of its major inconveniences is a weak failed calibration error signaling which subsequently may induce a high percentage of inadequate recording [40]. However, the integration into an anesthesia machine and the ease of use in many clinical situations is a strength.

### 7.4. Compressomyography 

This method is a user-friendly practical device that consists of a modified blood pressure cuff and two built-in electrodes. The method assesses quantitative neuromuscular blockade by calculating pressure changes emanating from muscle contraction within the cuff following peripheral nerve stimulation of the upper arm. This device is evaluated in a few studies; however, disagreement exists between results obtained for this device operating in the upper arm and others reflecting the adductor pollicis [41]. The technique is interesting and easy to set up, but more studies are necessary in the area of accuracy and precision of this device to adequately place this technique in the current clinical practice [42,43,44,45,46]. 

Most of these monitors available commercially are in agreement with each other on values near 90% TOFR but are usually in important disagreement for the onset of neuromuscular blockade and shallow blocks [47,48,49,50].

### 7.5. Techniques and Technologies Not Available for Clinical Use

#### 7.5.1. Mechanomyography 

MMG, which measures force generated by muscle contraction, is often called the gold standard of neuromuscular monitoring; however, despite its precision and accuracy, the cumbersome setup does not allow its use in routine clinical practice. The mechanomyograph needs a force transducer and a preload (around 300 g); therefore, the hand needs to be totally free, which makes it less suitable for most procedures. It also needs a stabilization period of 3–10 min, which is too long in routine clinical practice. Only one model is commercially available (Myograph 2000 (Biometer, Odense, Denmark)) for intraoperative monitoring [51].

#### 7.5.2. Phonomyography 

This technology is based on the principle that muscle contraction emits low-frequency sounds, which can be detected. The dedicated microphone is applied to the skin surface of the targeted muscle [18,52]. The intensity of the detected sound is proportional to the force of contraction; naturally, the signal needs to be cleaned from other noises. Intraoperative monitoring is reported for multiple peripheral muscles such as the adductor pollicis, corrugator supercilii and vastus medialis; for now, there is no commercially available monitor for routine use of this technology.

## 8. Current Trend

Based on the fact that quantitative neuromuscular block assessment is not performed by all anesthesia providers, a novel clinical score to estimate the risk of neuromuscular block has been suggested; this model includes liver failure, neurological disease, high neostigmine dose, metastatic tumor, female sex, short time elapsed between the last injection and extubation amino steroidal neuromuscular agent, high BMI (>35) absence of nurse anesthetist and an experienced surgeon was suggested to be superior to the intraoperative TOFR in predicting postoperative residual neuromuscular block [53], but this approach may no longer be justified, with many anesthetic societies already recommending systematic intraoperative monitoring [1,54,55]. In the 2022 ESAIC guidelines on perioperative management of neuromuscular blockade, three out of eight strong recommendations were about monitoring neuromuscular blockade, starting with a quantitative neuromuscular assessment of ulnar nerve stimulation, including the use of sugammadex for reversal of deep, moderate and shallow aminosteroid neuromuscular blockade, and finally recommending the reversal from neostigmine to be initiated only when TOF ratio is above 0.2. In addition, monitoring succinylcholine-induced paralysis and its recovery by quantitative neuromuscular monitoring is now also suggested in practice. In January 2023 the American Society of Anesthesiologists practice guidelines for monitoring and antagonism of neuromuscular blockade were also published, and a strong recommendation toward quantitative monitoring of intraoperative muscle relaxant administration was given [3]. 

Different techniques of monitoring are summarized in Table 2.

## 9. Monitoring Neuromuscular Blockade and Pharmacological Reversal

Most international guidelines recommend neuromuscular monitoring when administering pharmacological reversal. Current pharmacological reversals available are only neostigmine and sugammadex. Quantitative evaluation compared with qualitative evaluation or clinical assessment reduces the risk of residual paralysis.

### 9.1. Reversal with Neostigmine

Neuromuscular monitoring is essential during neostigmine reversal. In a recent meta-analysis of fourteen studies, neostigmine was reported to be effective in accelerating recovery in both types of non-depolarizing muscle relaxants with a limited incidence of side effects [56]. Neostigmine reversal should be used once the TOF ratio is >0.4 and less than 0.9 at a dose not exceeding 40 µg/kg; when the TOF ratio exceeds 0.6, 30 µg/kg should be appropriate [3]. When a dosage higher than 60 µg/kg was used, neostigmine was found to be an independent risk factor for postoperative pulmonary complications [57]. Spontaneous recovery should be allowed if TOFR is above 90%. When a simple nerve stimulator is present without quantitative neuromuscular monitoring, only 15 to 30 µg/kg is recommended since if a full dose of neostigmine is given, when the block is too shallow, neostigmine might have the opposite effect. Furthermore, it is not recommended to administer neostigmine when the expected waking time and extubation are longer than 10 min [3]. Unlike sugammadex, neostigmine reversal can be used in all types of non-depolarizing muscle relaxants; however, it is ineffective while administered during deep neuromuscular blockade.

### 9.2. Reversal with Sugammadex

While initially there were some thoughts that the reliable reversal with sugammadex would eliminate the need for the practice of intraoperative neuromuscular monitoring, it is now firmly believed that using sugammadex without monitoring could backfire as sugammadex can also reverse deep neuromuscular block with different higher dosages. Adequate monitoring would permit the adaptation and administration of the exact dose necessary and avoid inadequate reversal yielding residual paralysis Table 3. Sugammadex is only indicated with intermediate-acting aminosteroid relaxants, especially rocuronium. Sugammadex administration in appropriate dosage and time permits a reliable and rapid reversal [58]. In a retrospective analysis, Domenech et al. demonstrated that intraoperative monitoring in association with sugammadex would decrease the incidence of residual neuromuscular blockade [59].

## 10. Implementation of Neuromuscular Monitoring Culture 

It is almost generally accepted that quantitative neuromuscular monitoring should be performed in all patients having muscle relaxation. This objective starts with placing a quantitative monitoring device in each site providing anesthesia (traditional operating rooms and other non-operating rooms) and each post-anesthetic care unit. However, equipment alone is not enough; repeated educational efforts as part of a quality assurance program are mandatory [60]. Education of clinicians and other anesthesia providers on outcomes of untreated residual neuromuscular blockade [61], monitoring with indicators such as the use of neuromuscular monitoring whenever neuromuscular blockers are used, documenting and using the results to motivate anesthesia providers to pursue the trend, and elaborating clear protocols in the use of different antagonists would help to decrease the incidence of complications. 

## 11. Conclusions

With recent guidelines and protocols, all patients having muscle relaxants during anesthesia should be objectively monitored. A minimum recovery of TOFR at 90% is mandatory before extubation, and there is no justification to bypass this essential part of surveillance in anesthetized patients. 

## Figures and Tables

**Table 1:** Different types of stimulation.

Mode of Stimulation	Description	Observation
Train of four (TOF)	Supramaximal stimuli, frequency of 2 Hz, four stimuli (T1 to T4) every 0.5 ms, can be repeated every 10–15 s during induction and recovery and less frequently during maintenance, a fade (the fourth response weaker than the first one) is the sign of paralysis induced by NDMR.	Mostly used, in all situations. TOFR should be >0.9 before extubation
Post-tetanic count (PTC)	One 5 s tetanic (50 Hz) stimulus, followed 3 s later by 15 to 20 single twitch stimuli. The number of evoked responses obtained after this stimulation will predict the time of the reappearance of the first TOF evoked response.	For profound neuromuscular block, should be used only when TOF = 0
Double burst stimulation (DBS)	Two short bursts of three impulses of 0.2 ms. Each stimulation has a duration of 0.2 ms separated by 750 ms. A fade (second response weaker than the first one) appears when there is residual paralysis.	Useful for recovery, can detect a much lighter level of residual blockade, especially if no quantitative monitoring is used, but not as accurate as quantitative monitoring
Single twitch stimulation	A single repetitive supramaximal stimulation of 0.2 ms, a visual decrease in the evoked response means 75% of receptors are involved.	No more indications in routine clinical practice
Tetanic stimulation	A 50–200 Hz frequency stimulation for 5 s, can detect residual paralysis if there is any fade during stimulation.	Very painful, should never be used in awake patients, no real indication in routine clinical situations

**Table 2:** Summary of different types of monitoring.

Monitoring Technique	Principles	Observations
Acceleromyography	Measurement of acceleration, with a piezoelectric sensor.	Most used technique in routine practice, needs a free hand for calibration in most models. Baseline TOFR often >1, sometimes needing the normalization of values for recovery.
Kinemyography	Measurement of the electric signal yielded after a movement of a plastic sensor probe placed between the thumb and the index.	Easy to use. Results and recording less reliable, high percentage of auto-calibration failure.
Compressomyography	Stimulation of the brachial plexus, integrated into a noninvasive blood pressure cuff device, measuring the response in the arm muscles.	Very easy to use, no special setup necessary. Results are controversial, needs more studies.
Electromyography	Uses the measurement of the transcutaneous muscle action potential after nerve stimulation.	Fair results, may have interference with other devices. No need for a free hand.
Phonomyography	Measures the low-frequency sound emanating from muscle contraction.	No commercial device available. May be used in multiple sites.
Mechanomyography	Measures the evoked response of muscle contraction after nerve stimulation.	Not commercially available for routine practice, gold standard for research, cumbersome to install. Commercially not available.

**Table 3:** Sugammadex reversal and monitoring neuromuscular blockade.

Neuromuscular Monitoring	Aminosteroids and Sugammadex for Reversal
Upon administration of full dose, profound block, no PTC detectable	16 mg/kg
Deep block, PTC > 1	4–8 mg/kg
0 < TOF count < 3 (moderate block)	2 mg/kg
1 < TOF count < 4	2 mg/kg
TOF count = 4 TOF ratio < 0.9	2 mg/kg
TOF ratio = 0.9	Spontaneous recovery permitted

## Data Availability

Not applicable.
